# Macon Medical Society

**Published:** 1889-09

**Authors:** J. A. Toole

**Affiliations:** Rep.


### MACON MEDICAL SOCIETY.

July, 1889.

The President, Dr. N. G. Gewinner, in chair. Dr. H. McHatton, Secretary.

SUBJECT—NEW REMEDIES.

Dr. W. F. Holt said he had lately been using extensively in his practice Sulphonal, and found it a most excellent hypnotic. He had seen no bad effects from its use except in one case where he gave it very freely, and it produced double vision.

Dr. H. J. Williams concurred with Dr. Holt in his good opinion of Sulphonal, and said he had found it an excellent medicine in those cases where opium was objectionable on account of its nauseous taste and constipating effect; especially in the treatment of children.

Dr. H. McHatton said he had frequently given it in cases of *mania a potu*, and that the medicine seemed only adapted to cases of nervous irritation.

Dr. K. P. Moore said he could not say much with regard to Sulphonal, but was very familiar with antipyrine and could say for it that it promptly reduced the pulse and temperature, and seemed to have a very tranquilizing effect generally. That he often used it in nervous headache, and though in connection with other remedies he attributed a full share of the good effects obtained to antipyrine.

Dr. Williams did not think so well of it, and said he regarded it as a medicine that concealed the true condition of the patient, and that it was destined to fall into disuse.

Dr. R. O. Cotter said he had used it to some extent, but could testify to its pulse reducing qualities, as on himself he had given it a trial, and it reduced his to an alarming extent, besides producing other unpleasant symptoms.

Dr. H. J. Williams read a very interesting paper on pseudomembranous croup. The patient, a child recently from Deuran,

Mexico, was admitted into the orphans' home, and soon after became affected with the above disease, which from the first assumed a malignant form, the patient becoming cyano:ed and unconscious. At one time the dốctor thought that he would have to resort to tracheotomy and made arrangements to that effect, but by assiduous attentions, the observance of strict hygenic and curative measures, this last resort was obviated and the little sufferer recovered. During the treatment of the case a telegram from the neighborhood in Mexico, from which the child had come, was received to the effect that an epidemic of diphtheria was prevailing there. The mother of the child at the same time had a sore on her hand which the doctor regarded as diphtheritic, but yielded to treatment. There was no spread of the disease, and the doctor said there was one thing in the case he wished especially noticed, the violence of the case and the low temperature, ranging from 99 to 101 Fahrenheit.

Dr. Holt said he had seen the case and did not remember seeing but one of a similar character recover.

Dr. J. A. Tool said he had in his practice two similar cases, the first of which, not seen until the disease was far advanced, died; the second was given calomel at first, afterwards chlorate potassa, sulphur, freely applied to the affected membrane with inhalations of turpentine and lime, recovered.

Dr. W. C. Gibson said he had rather a strange case under his treatment, a woman who claimed to drink two common sized water buckets of water every 12 hours, and as he knew the patient he thought her statement true. The doctor had not yet satisfactorily examined the patient so as to account for her great thirst, and what became of the water, but said she was somewhat œdematous about her feet.

J. A. TOOLE, M. D., Rep.

